# Use of biologic or targeted-synthetic disease-modifying anti-rheumatic drugs and risk of diabetes treatment intensification in patients with rheumatoid arthritis and diabetes mellitus

**DOI:** 10.1093/rap/rkaa027

**Published:** 2020-06-22

**Authors:** Sarah K Chen, Hemin Lee, Yinzhu Jin, Jun Liu, Seoyoung C Kim

**Affiliations:** r1 Division of Pharmacoepidemiology and Pharmacoeconomics; r2 Division of Rheumatology, Immunology and Allergy, Brigham and Women’s Hospital, Harvard Medical School, Boston, MA, USA

**Keywords:** rheumatoid arthritis, biologic drug, diabetes

## Abstract

**Objectives:**

Given that RA treatment might affect the severity of diabetes mellitus (DM), we compared the risk of DM treatment intensification in patients with both RA and DM newly initiating a biologic DMARD or tofacitinib.

**Methods:**

Using claims data from the IBM MarketScan database (2005–2016), we identified patients aged ≥18 years with RA who initiated abatacept, a TNF inhibitor (TNFi), rituximab, tocilizumab or tofacitinib. Patients were required to have type 1 or type 2 DM and to use at least one antidiabetic drug at baseline. We assessed DM treatment intensification (i.e. addition of a new insulin or non-insulin antidiabetic medication). We also assessed non-insulin antidiabetic medication switching events.

**Results:**

We included 10 019 patients with RA and DM initiating a biologic DMARD or tofacitinib. Baseline insulin use was the highest in rituximab initiators (44%) and lowest in tofacitinib initiators (35%). The incidence rate per 1000 person-years for DM treatment intensification ranged from 148.2 (tofacitinib) to 198.0 (rituximab). The risk of DM treatment intensification was similar between abatacept and TNFi [hazard ratio (HR) 0.97, 95% CI: 0.82, 1.15], rituximab (HR 0.99, 95% CI: 0.79, 1.23) and tocilizumab (HR 0.94, 95% CI: 0.74, 1.19), but lower for tofacitinib compared with abatacept (HR 0.67, 95% CI: 0.50, 0.90). The risk of non-insulin DM treatment switching was not different between abatacept and other biologic DMARDs.

**Conclusion:**

In patients with both RA and DM, we found no difference in the risk of DM treatment switching or intensification after initiating abatacept *vs* TNFi, rituximab and tocilizumab, whereas the risk appeared to be lower for tofacitinib.

**Key messages**

- RA treatment may affect the severity of diabetes mellitus in patients who have both RA and diabetes mellitus.
- We noted no difference in the risk of diabetes mellitus treatment switching or intensification after initiating abatacept *vs* TNFi, rituximab and tocilizumab among patients with both RA and diabetes mellitus.
- The risk of diabetes mellitus treatment switching or intensification appeared to be lower for tofacitinib *vs* abatacept.

## Introduction

RA and diabetes mellitus (DM) are both associated with increased risk of cardiovascular disease, which is the leading cause of death for both RA and DM [1–5]. Patients with RA who have coexisting DM represent a subset of patients at an even higher cardiovascular disease risk [6]. Although the pathophysiology of type 1 (T1) DM and differs from that of type 2 (T2) DM, both conditions have possible pathophysiological associations with RA. Pro-inflammatory cytokines involved in RA disease pathogenesis are known to interfere with insulin-signalling pathways that are associated with T2DM [7, 8]. T1DM is an autoimmune disease characterized by immune-mediated destruction of insulin-producing β-cells in the pancreas. The two autoimmune diseases may be associated with each other owing to co-occurrence of genetic and environmental factors [9, 10].

However, previous epidemiological studies have demonstrated varying estimates of risk for DM in patients with RA [11–14]. This might be attributable to the heterogeneity of RA disease treatment, which has been shown to affect the risk of diabetes [15–17]. In particular, the use of abatacept has been shown to improve insulin resistance [18, 19] and is associated with lower risk of incident DM in RA patients compared with infliximab and etanercept [17]. Furthermore, in a previous study of patients with RA and DM, abatacept initiators had a lower risk of myocardial infarction and coronary revascularization compared with TNF inhibitor (TNFi) initiators [6]. Additionally, tocilizumab has been observed to decrease glycosylated haemoglobin (HbA_1c_) levels during treatment for RA, in both diabetic and non-diabetic patients [20], and was associated with improved HbA_1c_ levels with 3 months of treatment compared with TNFi in a retrospective observational study [21]. The JAK–STAT signalling pathway, which is inhibited by tofacitinib, is also implicated in both T1DM and T2DM, with effects on β-cell destruction and insulin sensitivity [22, 23]. However, comparative effects of the various DMARDs used in the treatment of RA on improvement or exacerbation of coexisting DM is not known.

Given that patients with both RA and DM represent a patient population subset with high cardiovascular disease risk, the objective of this study was to compare the rates and risk of worsening DM measured by DM treatment intensification in patients with RA and DM initiating a new biologic DMARD or tofacitinib compared with abatacept.

## Methods

### Data source

We conducted a retrospective cohort study using insurance claims from the IBM MarketScan Research Database (MarketScan) from the years 2005 to 2016. MarketScan contains comprehensive longitudinal health-care insurance claims of primarily working individuals and family members in the USA and includes patient-level information on demographics, hospital, emergency department and outpatient visits and pharmacy dispensing.

The study protocol was reviewed and approved by the Institutional Review Board of the Brigham and Women’s Hospital (protocol number: 2017P001342). Patient informed consent was not required because the database was de-identified.

### Cohort selection

We identified RA patients using at least two inpatient or outpatient International Classification of Diseases, Ninth Revision, Clinical Modification (ICD-9) or 10^th^ Revision (ICD-10) codes of RA, separated by 7–365 days, and required a new dispensing for abatacept, a TNFi (adalimumab, certolizumab, etanercept, golimumab or infliximab), rituximab, tocilizumab or tofacitinib on or after the second RA diagnosis date [24]. We defined the index date as the date of first study drug dispensing. We required ≥365 days of continuous enrolment before the index date. We excluded patients <18 years of age and patients with a history of malignancy. All patients were required to have a diagnosis of DM based on at least one T1DM or T2DM ICD-9 or ICD-10 code plus at least one anti-DM drug dispensing during the 365-day baseline period. We excluded patients with gestational DM at baseline.

### Outcome

The primary outcome was DM treatment intensification, defined as the addition of a new type of insulin medication (basal or bolus) or a new non-insulin antidiabetic medication without stopping baseline DM medication. Our secondary outcome was switching of a non-insulin antidiabetic medication, defined as the start of a new non-insulin antidiabetic medication and stopping the baseline DM medication. For example, if a patient on metformin at baseline started a new antidiabetic medication (e.g. rosiglitazone), but stopped metformin, this was considered a switch. Antidiabetic drugs and their classification are included in Supplementary Table S1, available at *Rheumatology Advances in Practice* online.

To validate our study findings, we conducted a sensitivity analysis using herpes zoster infection as a positive control outcome. Herpes zoster infection was defined as either an inpatient ICD-9 or ICD-10 code for herpes zoster or an outpatient ICD-9 or ICD-10 code for herpes zoster plus the use of an anti-viral medication (acyclovir, valacyclovir or famciclovir) within 7 days of the diagnosis code [25].

### Study follow-up

Follow-up time started from the day after the index date to the first occurrence of any of the following events: occurrence of outcome of interest; disenrolment from the insurance; end of the study period (31 December 2016); death; or discontinuation or switch of the index drug.

### Covariates

We collected covariates during the 365-day baseline period before and on the index date. We assessed patient demographics (age, sex and region of residence), co-morbidities, including hypertension, obesity, smoking, alcohol use, cardiovascular disease, heart failure, hyperlipidaemia, depression, chronic renal disease, chronic liver disease, pulmonary disease and hypothyroidism, and the Charlson co-morbidity score [26]. We assessed the use of antidiabetic medications as listed in Supplementary Table S1 (available at *Rheumatology Advances in Practice* online) and use of DMARDs and prior use of CSs, cumulative prednisone-equivalent dose over 365 days, NSAIDs, selective cox-2 inhibitors (coxibs), opioids, anti-hypertensives, statins, anti-platelet drugs, anticoagulants, diuretics, laboratory test ordered (HbA_1c_, creatinine, blood urea nitrogen test, ESR or CRP) and markers of health-care utilization (number of outpatient visits, visits to primary care providers, rheumatologist, endocrinologist, emergency department, hospitalizations and number of unique generic prescriptions dispensed).

### Statistical analyses

We calculated the incidence rate of outcome events per 1000 person-years in each cohort. We used unadjusted and multivariable Cox-proportional hazards models to estimate hazard ratios (HRs) and 95% CIs of DM treatment intensification and switching events among abatacept initiators (common reference) *vs* other DMARDs. The multivariable Cox model included demographics (age, sex and index calendar year), co-morbidities of renal failure, liver disease, T1DM, Chalson co-morbidity score, medication use (CSs, MTX, HCQ, statin, number of previous biologic DMARDs, antidiabetic drugs and insulin use), and health-care utilization (number of rheumatologist visits, endocrinologist visits and primary care physician visits). In the sensitivity analysis, we introduced a 30-day lag time after the index date, because changes to DM treatment shortly after index DMARD initiation might not be attributable to the effects of the DMARD.

For the positive control analysis, we calculated the incidence rate per 1000 person-years and estimated the HR (95% CI) of herpes zoster infection among abatacept *vs* the other DMARDs, because tofacitinib is known to increase the risk of herpes zoster more than other biologic DMARDs [27]. For the adjusted analysis, the multivariable Cox model included age, sex, index calendar year, CS use, renal failure, liver disease, number of previous DMARDs, MTX use, HCQ use, statin use, number of rheumatology visits, number of primary care physician visits, use of antiviral medications, zoster vaccination, Charlson co-morbidity score and T1DM at baseline. We conducted all analyses using SAS v.9.4 (SAS Institute, Cary, NC, USA).

## Results

**Fig. 1:**
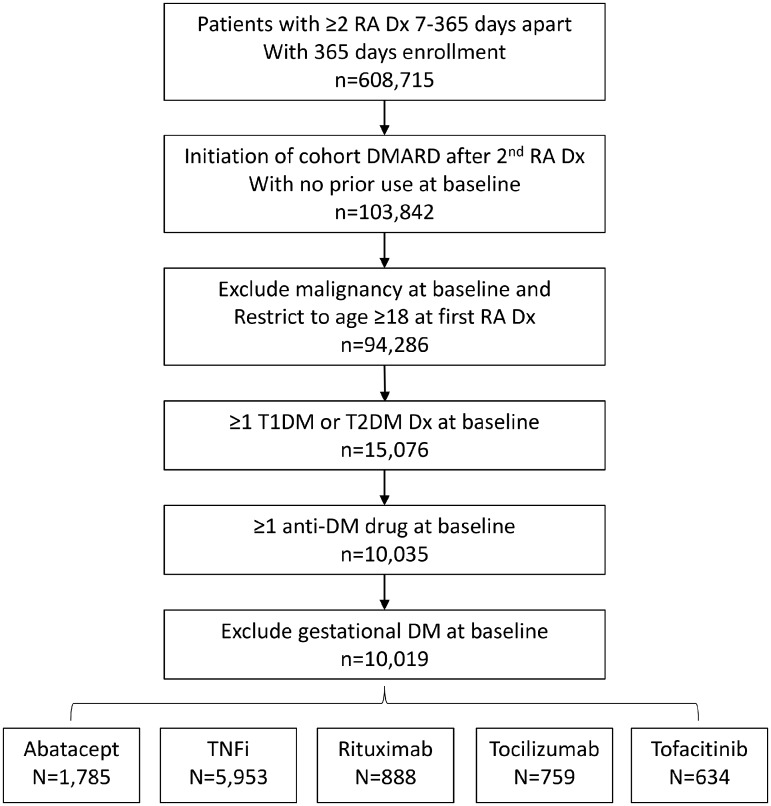
Flow diagram of cohort selection

We identified 10 019 patients with RA and DM who met the inclusion and exclusion criteria (Fig. 1). The mean age (s.d.) of the cohorts ranged from 56.71 (10.41) years (TNFi cohort) to 58.45 (10.26 years) (abatacept cohort), and between 71.5 and 78.9% were female (Table 1). The co-morbid conditions were lowest in the TNFi group and generally higher for the rituximab group, with a mean (s.d.) Charlson co-morbidity index score of 2.86 (1.16) for the TNFi cohort and 3.25 (1.46) for the rituximab cohort. Use of MTX or HCQ was most frequent in the TNFi cohort, and few patients in the TNFi group had used other biologic DMARDs or tofacitinib, whereas ≥41% of patients in the other cohorts had previously used TNFi (Table 2). More than 50% of patients used statins, and the rituximab cohort had the highest prevalence of any insulin use in the baseline period (44%).

**Table 1:** Baseline characteristics for patients with RA and diabetes mellitus by cohort status

Characteristic	Abatacept (*n* = 1785)	TNFi (*n* = 5953)	**ASMD** ^a^	Rituximab (*n* = 888)	**ASMD** ^a^	Tocilizumab (*n* = 759)	**ASMD** ^a^	Tofacitinib (*n* = 634)	**ASMD** ^a^
Age, mean (s.d.), years	58.45 (10.26)	56.71 (10.41)	0.17	58.09 (10.75)	0.03	57.61 (10.07)	0.08	58.03 (9.71)	0.04
Female, %	78.0	71.5	0.15	75.7	0.05	78.9	0.02	77.0	0.02
Type 1 DM, %	21.4	21.0	0.01	24.4	0.07	21.6	0.00	20.0	0.03
Co-morbid conditions									
Smoking, %	13.5	13.6	0.00	15.4	0.05	13.2	0.01	12.9	0.02
Obesity, %	18.4	18.5	0.00	21.0	0.07	24.9	0.16	25.2	0.17
Alcoholism, %	0.6	0.7	0.01	0.6	0.00	0.8	0.02	1.0	0.04
Hyperlipidaemia, %	57.3	56.9	0.01	58.3	0.02	65.2	0.16	67.4	0.21
Hypertension, %	70.3	67.2	0.07	72.4	0.05	72.6	0.05	73.5	0.07
Myocardial infarction, %	3.1	2.4	0.04	4.5	0.07	2.8	0.02	3.9	0.04
Heart failure, %	10.2	5.9	0.16	13.4	0.10	8.6	0.05	7.4	0.10
Cerebrovascular accident, %	4.2	3.6	0.03	5.4	0.06	5.3	0.05	4.3	0.00
Renal disease, %	9.4	7.5	0.07	12.1	0.09	10.8	0.05	11.2	0.06
Pulmonary disease, %	26.3	22.7	0.08	32.3	0.13	26.2	0.00	25.4	0.02
Liver disease, %	10.0	9.0	0.03	11.2	0.04	11.7	0.05	11.0	0.03
Hypothyroidism, %	21.2	19.5	0.04	22.4	0.03	24.1	0.07	27.6	0.15
Psychiatric disease, %	23.8	21.1	0.06	23.5	0.01	25.7	0.04	24.0	0.00
Co-morbidity index score, mean (s.d.)	3.06 (1.26)	2.86 (1.16)	0.17	3.25 (1.46)	0.14	3.05 (1.27)	0.01	3.07 (1.33)	0.01
Health-care utilization									
Ordered HbA_1c_, %	71.3	75.4	0.09	71.6	0.01	76.7	0.12	75.9	0.10
Ordered CRP, %	56.7	62.3	0.11	59.1	0.05	68.3	0.24	64.0	0.15
Ordered creatinine, %	82.1	85.0	0.08	85.3	0.09	87.0	0.14	83.8	0.05
ED visit, %	40.0	38.1	0.04	45.4	0.11	41.0	0.02	36.3	0.08
Hospitalization, %	25.1	20.8	0.10	34.0	0.20	21.6	0.08	18.5	0.16
PCP visits, *n*, mean (s.d.)	8.49 (10.15)	7.59 (8.01)	0.10	9.83 (12.25)	0.12	8.37 (9.61)	0.01	8.12 (9.83)	0.04
Rheumatologist visits, *n*, mean (s.d.)	4.51 (5.03)	3.32 (3.81)	0.27	4.54 (5.87)	0.01	5.73 (5.82)	0.22	4.31 (4.41)	0.04
Endocrinologist visits, *n*, mean (s.d.)	0.59 (1.97)	0.55 (1.69)	0.02	0.52 (1.61)	0.04	0.69 (1.62)	0.06	0.66 (1.61)	0.06
Any outpatient visits, *n*, mean (s.d.)	17.96 (9.54)	16.08 (8.76)	0.21	18.61 (10.21)	0.07	18.28 (9.08)	0.03	16.50 (8.50)	0.16
Prescription drugs, *n*, mean (s.d.)	21.04 (8.33)	19.79 (8.20)	0.15	21.14 (8.86)	0.01	21.23 (7.92)	0.02	20.81 (8.26)	0.03

a ASMD: absolute standardized mean difference, for which each drug group was compared with abatacept; DM: diabetes mellitus; ED: emergency department; HbA_1c_: glycosylated haemoglobin; PCP: primary care provider; TNFi: TNF inhibitor.

**Table 2:** Baseline medications for patients with RA and diabetes mellitus by cohort status

Medication	Abatacept (*n* = 1785)	TNF inhibitor (*n* = 5953)	**ASMD** ^a^	Rituximab (*n* = 888)	**ASMD** ^a^	Tocilizumab (*n* = 759)	**ASMD** ^a^	Tofacitinib (*n* = 634)	**ASMD** ^a^
Use of DMARDs^b^									
Abatacept, %	0	2.9	0.24	16.9	0.64	31.5	0.96	18.9	0.68
TNF inhibitor, %	59.9	0	1.73	41.8	0.37	51.0	0.18	44.6	0.31
Rituximab, %	3.8	0.7	0.21	0	0.28	7.4	0.16	6.5	0.12
Tocilizumab, %	3.1	0.8	0.17	6.2	0.15	0	0.25	12.3	0.35
Tofacitinib, %	2.0	0.5	0.14	2.5	0.03	5.0	0.16	0	0.20
HCQ, %	23.0	25.7	0.06	22.8	0.00	21.5	0.04	20.8	0.05
MTX, %	55.5	71.0	0.33	51.4	0.08	53.2	0.05	50.6	0.10
LEF, %	21.0	16.1	0.13	18.1	0.07	21.0	0.00	23.0	0.05
SSZ, %	8.9	11.1	0.07	7.2	0.06	8.4	0.02	8.5	0.01
Other DMARD, %	8.1	5.1	0.12	14.0	0.19	9.6	0.05	7.3	0.03
Previous use of biologic DMARD, *n*, mean (s.d.)	0.81 (0.70)	0.05 (0.24)	1.45	0.73 (0.72)	0.11	1.04 (0.69)	0.33	0.93 (0.77)	0.16
Use of oral glucocorticoid									
Within 30 days, %	43.8	39.8	0.08	52.6	0.18	44.3	0.01	45.6	0.04
Within 365 days, %	70.2	67.2	0.06	76.9	0.15	75.0	0.11	72.7	0.06
Cumulative prednisone equivalent dose in 365 days, mean (s.d.) in mg	1600.3 (6320.2)	1248.5 (5280.5)	0.06	2135.0 (4324.2)	0.10	1928.1 (4174.8)	0.06	1604.0(4694.9)	0.00
Use of antidiabetics									
Biguanides, %	64.5	68.2	0.08	59.2	0.11	63.6	0.02	67.4	0.06
Sulfonylurea, %	31.9	32.3	0.01	32.4	0.01	28.3	0.08	29.2	0.06
Glitazones, %	12.9	14.8	0.06	13.7	0.02	8.8	0.13	5.5	0.26
DPP-4 inhibitor, %	15.4	14.6	0.02	13.1	0.07	19.0	0.10	17.5	0.06
GLP-1, %	8.7	7.5	0.04	7.0	0.06	11.9	0.11	11.5	0.09
SGLT-2, %	2.1	2.4	0.02	1.8	0.02	3.3	0.07	5.5	0.18
Meglitinides, %	1.9	1.8	0.01	1.6	0.02	1.7	0.02	2.2	0.02
AGI, %	0.4	0.4	0.00	0.2	0.04	0.4	0.00	0.3	0.02
Amylin analogue, %	0.3	0.4	0.02	0.2	0.02	0.4	0.02	0.3	0.00
Insulin, any, %	39.4	36.4	0.06	44.1	0.10	42.7	0.07	35.0	0.09
Insulin, bolus, %	26.7	25.2	0.03	32.0	0.12	28.3	0.04	24.0	0.06
Insulin, basal, %	29.9	26.1	0.08	32.2	0.05	31.6	0.04	25.2	0.11
Insulin, mixed, %	2.0	2.1	0.01	2.0	0.00	1.6	0.03	0.8	0.10
Other medications									
Statins, %	57.2	56.7	0.01	54.2	0.06	60.9	0.08	62.2	0.10
ACE/ARB, %	65.1	65.7	0.01	65.7	0.01	63.5	0.03	68.9	0.08
β-Blockers, %	32.0	28.1	0.09	33.9	0.04	33.1	0.02	34.9	0.06
Calcium channel blockers, %	25.9	25.0	0.02	25.7	0.00	24.4	0.03	25.4	0.01
Coxib, %	10.0	11.4	0.05	11.4	0.05	10.0	0.00	8.7	0.04
NSAIDs, %	45.0	55.1	0.20	39.9	0.10	47.0	0.04	45.9	0.02
Anticoagulants, %	9.0	6.4	0.10	11.4	0.08	9.2	0.01	6.9	0.08
Antiplatelets, %	10.4	10.2	0.01	11.3	0.03	8.7	0.06	10.4	0.00
Diuretics, %	51.7	48.7	0.06	51.5	0.00	53.5	0.04	50.0	0.03
Opioids, %	80.0	75.7	0.10	81.4	0.04	80.4	0.01	76.7	0.08

DPP-4: dipeptidyl peptidase-4 inhibitor GLP-1: glucagonlike peptide–1 (GLP-1) agonists SGLT-2: sodium-glucose co-transporter-2 inhibitors AGI: alpha-glucosidase inhibitors ACE/ARB: angiotensin-converting enzyme inhibitors/angiotensin II receptor blockers Coxib: selective cox-2 inhibitors. ^a^ASMD: absolute standardized mean difference, for which each drug group was compared with abatacept. ^b^Prior use of DMARDs was assessed during 365 days before the index date (excluding the index date).

During a mean follow-up of 9 months on treatment with biologic DMARDs or tofacitinib, there were 1399 total DM treatment intensification events in all cohorts (Table 3). The incidence rate of DM treatment intensification was highest in the rituximab cohort (198.0 per 1000 person-years, 95% CI: 166.0, 236.1) and lowest in the tofacitinib group (148.2 per 1000 person-years, 95% CI: 114.6, 191.7). Insulin intensification events were highest in the tocilizumab group (incidence rate 83.2 per 1000 person-years, 95% CI: 62.2, 111.5) and lowest in the tofacitinib group (incidence rate 60.9 per 1000 person-years, 95% CI: 41.2, 90.2). Non-insulin DM treatment intensification events were also lowest in the tofacitinib group (incidence rate 82.3 per 1000 person-years, 95% CI: 58.5, 115.7).

**Table 3:** Risk of diabetes treatment intensification and switching in study cohort initiating DMARD therapy

Event	Patients (*n*)	Events (*n*)	Person-years (*n*)	Incidence rate (95% CI)	HR_1_ (95% CI)	HR_2_ (95% CI)
All intensification events (insulin and non-insulin)						
Abatacept	1785	248	1264.3	196.2 (173.2, 222.2)	1.0 (reference)	1.0 (reference)
TNF inhibitors	5953	875	4719.0	185.4 (173.5, 198.1)	0.99 (0.86, 1.14)	0.97 (0.82, 1.15)
Rituximab	888	124	626.4	198.0 (166.0, 236.1)	1.00 (0.81, 1.24)	0.99 (0.79, 1.23)
Tocilizumab	759	94	514.6	182.7 (149.2, 223.6)	0.93 (0.73, 1.18)	0.94 (0.74, 1.19)
Tofacitinib	634	58	391.4	148.2 (114.6, 191.7)	0.72 (0.54, 0.96)	0.67 (0.50, 0.90)
Insulin intensification						
Abatacept	1785	102	1356.6	75.2 (61.9, 91.3)	1.0 (reference)	1.0 (reference)
TNF inhibitors	5953	320	5152.9	62.1 (55.7, 69.3)	0.86 (0.69, 1.07)	0.87 (0.67, 1.14)
Rituximab	888	50	673.2	74.3 (56.3, 98.0)	0.98 (0.70, 1.37)	0.90 (0.64, 1.27)
Tocilizumab	759	45	540.6	83.2 (62.2, 111.5)	1.10 (0.77, 1.56)	1.14 (0.80, 1.64)
Tofacitinib	634	25	410.3	60.9 (41.2, 90.2)	0.78 (0.50, 1.20)	0.83 (0.52, 1.30)
Non-insulin intensification						
Abatacept	1785	146	1326.6	110.1 (93.6, 129.4)	1.0 (reference)	1.0 (reference)
TNF inhibitors	5953	558	4936.4	113.0 (104.0, 122.8)	1.08 (0.90, 1.30)	1.05 (0.84, 1.31)
Rituximab	888	75	656.4	114.3 (91.1, 143.3)	1.03 (0.78, 1.36)	1.06 (0.80, 1.41)
Tocilizumab	759	50	540.1	92.6 (70.2, 122.2)	0.84 (0.61, 1.16)	0.83 (0.60, 1.15)
Tofacitinib	634	33	401.1	82.3 (58.5, 115.7)	0.70 (0.48, 1.02)	0.59 (0.40, 0.87)
Non-insulin switching						
Abatacept	1,503	84	1157.7	72.6 (58.6, 89.9)	1.0 (reference)	1.0 (reference)
TNF inhibitors	5050	320	4387.9	72.9 (65.4, 81.4)	1.04 (0.82, 1.33)	1.02 (0.77, 1.34)
Rituximab	701	48	526.8	91.1 (68.7, 120.9)	1.22 (0.85, 1.73)	1.20 (0.84, 1.72)
Tocilizumab	625	35	445.3	78.6 (56.4, 109.5)	1.08 (0.73, 1.60)	1.05 (0.70, 1.58)
Tofacitinib	536	26	345.7	75.2 (51.2, 110.5)	0.99 (0.64, 1.54)	1.04 (0.66, 1.64)

Incidence rate is per 1000 person-years. HR_1_: hazard ratio 1, unadjusted Cox model. HR_2_: hazard ratio 2, Cox model adjusted for age, sex, index year, CS use, renal failure, liver disease, number of previous biologic DMARDs, MTX use, HCQ use, statin use, number of oral antidiabetic drugs, number of insulin drugs, number of rheumatologist visits, number of primary care physician visits, number of endocrinologist visits, Charlson co-morbidity score and type 1 diabetes at baseline.

In multivariable-adjusted analysis, there was no difference in the risk of DM treatment intensification between abatacept, TNFi, rituximab and tocilizumab. However, the risk of DM treatment intensification was lower in the tofacitinib cohort (HR 0.67, 95% CI: 0.50, 0.90) compared with abatacept. Much of the lower risk appeared to be driven by non-insulin DM treatment intensification events for tofacitinib compared with abatacept (HR 0.59, 95% CI: 0.40, 0.87). There were no differences in the rates and risk of non-insulin DM treatment switching events across all biologic DMARDs and tofacitinib. Results remained similar in sensitivity analysis when we introduced a 30-day lag time after the index date (Supplementary Table S2, *Rheumatology Advances in Practice* online).

To support the findings of our study, we assessed a positive control outcome of herpes zoster infection and found a >2-fold higher risk with tofacitinib compared with abatacept (Table 4).

**Table 4:** Risk of herpes zoster infection in study cohort initiating DMARD therapy: positive control analysis

DMARD	Patients (*n*)	Events (*n*)	Person- years (*n*)	Incidence rate (95% CI)	HR_1_ (95% CI)	HR_2_ (95% CI)
Abatacept	1785	24	1399.1	17.2 (11.5, 25.6)	1.0 (reference)	1.0 (reference)
TNF inhibitors	5953	93	5316.9	17.5 (14.3, 21.4)	1.03 (0.66, 1.61)	1.48 (0.88, 2.49)
Rituximab	888	23	691.2	33.3 (22.1, 50.1)	1.93 (1.09, 3.42)	1.82 (1.02, 3.24)
Tocilizumab	759	19	557.1	34.1 (21.8, 53.5)	1.99 (1.09, 3.63)	1.98 (1.06, 3.68)
Tofacitinib	634	15	408.2	36.8 (22.2, 61.0)	2.12 (1.11, 4.05)	2.16 (1.09, 4.28)

Incidence rate is per 1000 person-years. HR_1_: hazard ratio 1, unadjusted Cox model. HR_2_: hazard ratio 2, Cox model adjusted for age, sex, index year, CS use, renal failure, liver disease, number of previous biologic DMARDs, MTX use, HCQ use, statin use, number of oral antidiabetic drugs, number of insulin drugs, number of rheumatologist visits, number of primary care physician visits, antiviral medication use, zoster vaccination, Charlson co-morbidity score and type 1 diabetes at baseline.

## Discussion

In this study of 10 019 patients with RA and DM who were newly initiating a biologic DMARD or tofacitinib, we found no difference in the risk of DM treatment intensification between abatacept and other biologic DMARDs but observed a lower risk of DM treatment intensification in tofacitinib initiators *vs* abatacept. The risk of DM treatment intensification (both insulin and non-insulin) was 33% lower risk for tofacitinib initiators compared with abatacept, and the risk of non-insulin DM medication intensification was 41% lower for tofacitinib. Non-insulin DM medication switching events were similar for all biologic DMARDs and tofacitinib.

Although the effect of DMARDs on control of coexisting DM in patients with RA and DM has not been reported, we had expected to find the lowest rates and risk of DM treatment intensification with abatacept use. In a large cohort study of RA patients, incident DM risk was lower in abatacept initiators compared with infliximab or etanercept initiators [17]. In mouse models of obesity, abatacept was shown to improve insulin resistance [28], and small case report and observational studies have suggested an insulin sensitizing effect of abatacept in patients with RA [18, 19, 29]. A proposed mechanism suggests that inhibiting T cell costimulation reduces the effect of T cells in adipose tissues and improves regulatory T cell function, which are thought to improve insulin sensitivity [19]. Furthermore, abatacept use in patients with T1DM has been shown to delay the decline of β-cell function [30, 31]. Likewise, there have been observational reports of improved HbA_1c_ with tocilizumab use in patients with RA [20, 21].

Based on these previous findings, we had expected to find a lower risk of DM treatment intensification with abatacept and/or tocilizumab but instead identified a lower risk of DM treatment intensification with tofacitinib compared with abatacept. To validate this finding, we ran a sensitivity analysis using herpes zoster infection as a positive control outcome in our cohort and found a high risk of herpes zoster infection associated with tofacitinib and other biologics compared with abatacept, as previous studies reported in the literature [25]. Although there are no previous studies that have looked at the effect of tofacitinib or JAK inhibition on DM, the JAK–STAT pathway is implicated in both T1 and T2DM, which might explain the findings of our study. The JAK1/2–STAT1 pathway is involved in the pro-inflammatory cytokine production that leads to β-cell destruction in T1DM [22, 32–34]. In T2DM, dysregulation of the leptin-induced JAK–STAT signalling pathway is thought to play a key role in insulin resistance [23]. However, no studies have examined the effect of JAK inhibition on DM directly. A large observational study comparing risk of incident DM among 67 756 RA patients with biologic or targeted synthetic DMARDs has shown that abatacept was associated with a lower risk of DM compared with infliximab or adalimumab; however, the effect estimate for tofacitinib was highly imprecise owing to the small sample size in the tofacitinib group [17]. Although we have more solid evidence for abatacept showing a beneficial effect on T1 and T2DM, existing evidence for tofacitinib is limited to support our study finding. We have observed high proportion of oral glucocorticoids (>70%) at baseline. Although use of glucocorticoids leads to worsening of T2DM, thus requiring more DM treatment intensification, we have adjusted for baseline use of glucocorticoids in the adjusted Cox regression model.

Both RA and DM are chronic conditions that constitute excess cardiovascular risk compared with the general population, with a high burden of medication use for disease management. Furthermore, the presence of both these diseases in an individual constitutes a condition of multimorbidity, which adds to the complexity of treating two conditions that are already challenging to manage. In women with RA, multimorbidity has recently been shown to be associated with cardiovascular mortality [35] and might play a role in the excess cardiovascular risk seen in patients with RA. Although many DMARD options are now available for RA treatment, how these drugs might affect coexisting conditions has not been studied extensively. In addition to the potential sequelae of poor glycaemic control, worsening DM can lead to the addition of a new antidiabetic medication, including insulin, which significantly impacts the quality of life in a patient population with a high burden of polypharmacy. We conducted a large real-world-based cohort study, including 10 019 patients with both RA and DM, to study the effect of RA treatment on DM as multimorbid conditions, which is an important area of research that is poorly understood at present.

This study has limitations. Although we stratified the outcome of DM medication intensification by insulin *vs* non-insulin DM medications, we did not examine dose escalations of the same baseline drugs. The prescribed dose and number of days of supply are available in pharmacy claims but have limitations in accuracy to assess the real dose, because in practice patients often adjust their antidiabetic medication dosing without a new prescription, particularly for insulin. Although it is possible to use methods to control for this by requiring a standard follow-up time to obtain a better estimate of the real amount and dosing of medications, as in previous studies [36], we were limited by the overall short follow-up time (mean follow-up of 0.75 years) available in our cohorts. In addition, there might be covariate misclassifications and unmeasured confounding, including baseline disease severity and duration for RA and DM.

In conclusion, there was no difference in the risk of DM treatment intensification in patients with RA and DM initiating a TNFi, rituximab or tocilizumab *vs* abatacept, but the risk was lower for tofacitinib initiators compared with abatacept initiators. Although this finding might be related to the effect of JAK–STAT pathway inhibition on mechanisms involved in insulin resistance and β-cell dysfunction in DM, further studies are needed to reproduce these findings and shed light on potential underlying mechanisms.

*Funding*: This study was supported by an investigator-sponsored research grant from Bristol-Myer-Squibb. The funding source played no role in the study design, data analysis or interpretation of data or presentation of results. The funder was given the opportunity to make non-binding comments on a draft of the manuscript, but the authors retained the right of publication and determined the final wording.

*Disclosure statement*: S. C. Kim has received research grants from the Brigham and Women’s Hospital from Pfizer, AbbVie, Bristol-Myers Squibb, and Roche for other studies. The other authors have declared no conflicts of interest.

## Supplementary Material

rkaa027_Supplementary_Data
